# Anxiety, depression, health-related quality of life, and mortality among colorectal patients: 5-year follow-up

**DOI:** 10.1007/s00520-022-07177-1

**Published:** 2022-06-23

**Authors:** Miren Orive, Ane Anton-Ladislao, Santiago Lázaro, Nerea Gonzalez, Marisa Bare, Nerea Fernandez de Larrea, Maximino Redondo, Amaia Bilbao, Cristina Sarasqueta, Urko Aguirre, José M. Quintana, for the REDISSEC-CARESS/CCR group

**Affiliations:** 1 Departamento Psicología Social. Facultad Farmacia, UPV/EHU, Vitoria, Spain; 2 Unidad de Investigación, Hospital Galdakao-Usansolo, Galdakao, Bizkaia Spain; 3 Servicio de Cirugía General, Hospital Basurto, Bilbao, Bizkaia Spain; 4 Unidad de Epidemiología Clínica, Corporacio Parc Tauli, Barcelona, Spain; 5 Centro Nacional de Epidemiología, ISCIII, Madrid, Spain; 6 Unidad de Investigación, Hospital Costa del Sol, Malaga, Spain; 7 Unidad de Investigación, Hospital Basurto, Bilbao, Bizkaia Spain; 8 Unidad de Investigación, Hospital Donostia/BioDonostia, Donostia, Guipuzkoa Spain; 9 Red de Investigación en Servicios Sanitarios y Enfermedades Crónicas (REDISSEC), Galdakao, Spain; 10 CIBER Epidemiología y Salud Pública (CIBERESP), Madrid, Spain; 11 Red de Investigación en Cronicidad, Atención Primaria y Promoción de la Salud (RICAPPS), Bizkaia, Spain

**Keywords:** Colorectal cancer, Patient-reported outcome measures, Health-related quality of life, Cohort studies, Longitudinal studies

## Abstract

**Purpose:**

Health-related quality of life (HRQoL) measurement represents an important outcome in cancer patients. We describe the evolution of HRQoL over a 5-year period in colorectal cancer patients, identifying predictors of change and how they relate to mortality.

**Methods:**

Prospective observational cohort study including colorectal cancer (CRC) patients having undergone surgery in nineteen public hospitals who were monitored from their diagnosis, intervention and at 1-, 2-, 3-, and 5-year periods thereafter by gathering HRQoL data using the EuroQol-5D-5L (EQ-5D-5L), European Organization for Research and Treatment of Cancer’s Quality of Life Questionnaire-Core 30 (EORTC-QLQ-C30), and Hospital Anxiety and Depression Scale (HADS) questionnaires. Multivariable generalized linear mixed models were used.

**Results:**

Predictors of Euroqol-5D-5L (EQ-5D-5L) changes were having worse baseline HRQoL; being female; higher Charlson index score (more comorbidities); complications during admission and 1 month after surgery; having a stoma after surgery; and needing or being in receipt of social support at baseline. For EORTC-QLQ-C30, predictors of changes were worse baseline EORTC-QLQ-C30 score; being female; higher Charlson score; complications during admission and 1 month after admission; receiving adjuvant chemotherapy; and having a family history of CRC. Predictors of changes in HADS anxiety were being female and having received adjuvant chemotherapy. Greater depression was associated with greater baseline depression; being female; higher Charlson score; having complications 1 month after intervention; and having a stoma. A deterioration in all HRQoL questionnaires in the previous year was related to death in the following year.

**Conclusions:**

These findings should enable preventive follow-up programs to be established for such patients in order to reduce their psychological distress and improve their HRQoL to as great an extent as possible.

**ClinicalTrials.gov Identifier:**

NCT02488161

**Supplementary Information:**

The online version contains supplementary material available at 10.1007/s00520-022-07177-1.

## Introduction

Colorectal cancer (CRC) is the third most common cancer among men and the second among women and its incidence continues to increase worldwide. [1] At the same time, thanks to advancements in screening, surgical techniques, and multimodal therapy, the proportion of patients surviving CRC has grown over the past 20 years. [2] Five-year survival of patients diagnosed with localized disease exceeds 85%. [3]

With these improvements in survivorship and an aging population, understanding and improving health-related quality of life (HRQoL) is becoming an important field of research. [1, 4] Assessment of HRQoL might support the choice and design of appropriate interventions and survivorship care plans. [3, 4] HRQoL in CRC survivors has been addressed in prior studies, but, as mentioned by Moserh et al. (2016) [5] in a recent review, many of these studies were cross-sectional, [5–8] dealt with the effects of treatment in the short term (less than 2 years), [4, 5, 8] did not address the impact of real baseline outcomes on prognosis (most studies enrolled the cohort after the start of active treatment), [9, 10] relied on relatively small sample sizes or on a small response rate, [4, 11] or only used general HRQoL questionnaires. [3, 10]

In addition, HRQoL is emerging as not only an important outcome in survivorship care, but also as a factor, that influences mortality. [12] In patients with CRC, higher HRQoL has been associated with a lower risk of dying. [3, 4] However, the underlying mechanisms of this association are not entirely clear, either because prior studies primarily assessed HRQoL in patients with advanced stages of the disease [3] or because they did not monitor tumor stage, recurrence, or patients’ comorbidities. [4]

In our study, we try to overcome these limitations. The goals of this study were:

1. To compare the evolution of anxiety and depression domains of the HADS questionnaire and HRQoL among CRC survivors with that of the normative population;
2. To prospectively assess the course of symptoms of anxiety, depression, and HRQoL over a 5-year period in relation to mortality; and
3. To identify risk factors related to losing HRQoL or increasing anxiety or depression during that follow-up period

## Material and methods

### Patients

This is a longitudinal prospective observational analytic cohort study, which includes patients from nineteen public hospitals representing nine provinces in Spain, all of which operate under the Spanish National Health Service (SNHS), which is responsible for the majority of the national population, with a planned patient follow-up period of 5 years. Patients with colon or rectal cancer scheduled to undergo surgery between June 2010 and December 2012 were informed of the goals of the study and were invited to participate. To enroll in the study the patients should written informed consent. The study was approved by the Basque Ethics Committee (Approval Number: 11/23/2010) and all study data were kept confidential. Patients were deemed eligible for this study if they were on the surgical waiting list of one of the participating hospitals and had a diagnosis of surgically resectable colon or rectal cancer. Exclusion criteria were in situ cancer, an unresectable tumor, terminal disease, inability to respond to questionnaires for any reason, and any severe mental or physical conditions that might prevent the patient from responding to questionnaires, as well as failure to consent to participate. Detailed information of the protocol has been published elsewhere. [13]

### Data collection

Clinical data and patient-reported outcome measures (PROMs) were collected at baseline time (before surgery), and at 1, 2, 3, and 5 years after surgery. Data collected at hospital admission included sociodemographic data, clinical data (including information about comorbidities based on the Charlson Comorbidity Index), [14, 15] preoperative data, outpatient anesthesia data related to the surgical intervention, American Society of Anesthesiologists (ASA) class [16] pathology data including TNM (Tumor-Node-Metastasis categories) stage, infiltrated lymph nodes, and data related to the period of admission after surgery (including the presence of complications, need for reoperation, readmission, and death). Clinical data were gathered from medical records and databases by qualified reviewers. Instruction manuals were prepared to guide the data collection process, in order to ensure consistency among centers and reviewers.

Patients completed Spanish versions of the following patient-reported outcome measures (PROMs):

#### Symptoms of anxiety and depression

The Hospital Anxiety and Depression Scale (HADS) [13, 17] was used as a specific questionnaire to measure symptoms of anxiety and depression in individuals with a physical illness. This is a 14-item measure: seven items for depressive symptoms and seven items for anxiety. A subscale score of 0 to 7 indicates the absence of anxiety or depression; 8 to 10 a possible case of anxiety or depression; and 11 or higher a probable case of anxiety or depression.

#### Health-related quality of life

The European Organization for Research and Treatment of Cancer’s Quality of Life Questionnaire Core 30 (EORTC-QLQ-C30) [3, 18] is a health-related quality of life-specific questionnaire widely used in cancer research. It consists of 30 items that assess five functioning domains, eight cancer symptom domains, financial difficulties, and global quality of life. Scores were transformed to a 0-to-100 scale, with a high score on the Functional scale and on Global Quality of Life scale indicating better functioning and HRQoL. For the symptom domains, higher scores indicate a greater symptom burden. The EuroQol-5D-5L (EQ-5D-5L) [19, 20] is a generic HRQoL measure which consists of two parts: (a) the descriptive system comprises five-level Likert-type dimensions (mobility, self-care, usual activities, pain/discomfort, and anxiety/depression) with five answer options defining different levels of severity. Combining these five dimensions, it can be obtained multiple health states and a weighted health score denominated utility index, which is associated with each health state. This index ranges from negative values (0 is equivalent to death and negative values correspond to those less preferred states than death by general population) to 1 (perfect health). And the visual analogue scale (VAS) records the respondent’s self-rated health on a vertical, visual analogue scale, ranging from 0 (worst imaginable state of health) to 100 (best imaginable state of health). For this study, only the descriptive system was taken into account.

Normative data were obtained from Waldman et al. (2013) [21] for the EORTC-QLQ-C30; Hernandez et al. (2018) [20] for the EQ-5D-5L; and Hinz et al. (2011) [22] for the HADS. The normative population data obtained for the EQ-5D is from Spain, but we were unable to find such data for the HADS and EORTC, and so a German sample was used instead.

### Statistical analysis

The main outcomes of this study were changes in the Hospital Anxiety and Depression Scale, the EuroQol-5d, and the EORTC questionnaires over the 5-year follow-up period. All scales were measured at baseline and at four points in time (1, 2, 3, and 5 years) after the intervention. All were assessed at each measurement point as a continuous variable. For exploratory analysis, a stratified analysis was performed, using means and standard deviations and according to patients’ vital status throughout the evolution (patients dying in the first year following the intervention, patients dying from 1 to 2 years, patients dying from 2 to 3 years, patients dying from 3 to 5 years, and patients alive at 5 years of follow-up). The results were summarized in various line charts, where the value in each of the questionnaires of the normal population was shown with a red line. Statistical significance with regard to values of the normal population was assessed by confidence intervals (95% CI) of the descriptive parameters. Parameters with *p*<0.20 in the univariable analysis were entered into multivariable generalized linear mixed models to assess the effects of the independent variables on the main outcomes. The independent variables included were age, gender, Charlson’s index, and TNM stage, as well as other clinical and social characteristics aforementioned. In these models, random intercepts and unstructured variance-covariance matrix were used. *R*-squared was used to assess the proportion of explained variance for a dependent variable, by independent variables of the models.

Statistical significance was assumed when *p*<0.05. All statistical analyses were performed using SAS 9.4, and figures were developed by R v.3.5.2.

## Results

### Sample characteristics

A flowchart describing the cohort evolution during the 5 years of follow-up is included in Supplementary Figure 1. Table 1 shows the characteristics of the sample and differences between surviving and non-surviving patients. At 5 years post-intervention nearly 30% of the patients had died. CRC survivors in this study had a mean age of 67 at diagnosis, younger than non-survivors (72 years), and enjoyed better health (fewer comorbidities: 18% vs 33% have ≥4 on the Charlson Comorbidity Index), with less severe TNM stages (4% vs 22% with TNM IV), fewer complications on admission (40% vs 53%) and at the first month following surgery (17% vs 22%), less urgent surgery (2% vs 7%), and less need for help at home at baseline (49% vs 34% did not require help).

**Table 1:** Demographic and clinical characteristics of patients included in the study

	Total group *N* (%)	Survivors^a^ *N* (%)	Non-survivors *N* (%)	*p*-value
Total	2531	1780 (70.33)	751 (29.67)	
Sex (man)	1603 (61.63)	1097 (61.63)	506 (67.38)	0.0061
Age*	68.46 (11.01)	67.01 (10.82)	71.91 (10.68)	<0.0001
Charlson Comorbidity index (≥ 4)	573 (22.64)	323 (18.15)	250 (33.29)	<0.0001
pTNM^b^				<0.0001
0,I,II	1447 (57.49)	1167 (65.75)	280 (37.74)	
III	828 (32.90)	533 (30.03)	295 (39.76)	
IV	242 (9.61)	75 (4.23)	167 (22.51)	
Adjuvant chemotherapy (yes)	1189 (47.96)	836 (46.99)	353 (50.43)	0.1232
Neoadjuvant chemotherapy (yes)	389 (15.69)	279 (15.68)	110 (15.71)	0.9846
Complications during admission (yes)	1105 (43.66)	704 (39.55)	401 (53.40)	<0.0001
Infectious	525 (20.74)	311 (17.47)	214 (28.50)	<0.0001
Surgical	341 (13.47)	215 (12.08)	126 (16.78)	0.0016
Medical	540 (21.34)	319 (17.92)	221 (29.43)	<0.0001
Complications within 1 month (yes)	463 (18.39)	302 (16.97)	161 (21.85)	0.0040
Infectious	170 (6.75)	108 (6.07)	62 (8.41)	0.0329
Surgical	149 (5.92)	106 (5.96)	43 (5.83)	0.9071
Medical	86 (3.42)	45 (2.53)	41 (5.56)	0.0001
Family history of neoplasia (yes)	868 (37.99)	651 (40.23)	217 (32.53)	0.0006
Family history of colorectal cancer (yes)	208 (8.22)	156 (8.76)	52 (6.92)	0.1236
Type of surgery				<0.0001
Programmed	2440 (96.40)	1744 (97.98)	696 (92.68)	
Urgent	91 (3.60)	36 (2.03)	55 (7.32)	
Social support				<0.0001
Does not need help	918 (44.26)	715 (48.51)	203 (33.83)	
Needs and does not receive help	23 (1.11)	18 (1.22)	5 (0.83)	
Receives help	1133 (54.63)	741 (50.27)	392 (65.33)	
Mortality				<0.0001
During admission	40 (1.58)	0 (0)	40 (5.33)	
Within 1 month after admission	9 (0.36)	0 (0)	9 (1.20)	
1 month–1 year after admission	148 (5.85)	0 (0)	148 (19.71)	
1–2 years after admission	154 (6.08)	0 (0)	154 (20.51)	
2–3 years after admission	146 (5.77)	0 (0)	146 (19.44)	
3–5 years after admission	254 (10.04)	0 (0)	254 (33.82)	
5-year survivors	1780 (70.33)	1780 (100)	0 (0)	

*N*, frequencies; %, percentages

*Mean (standard deviation)

^a^Survivors: 5-year survivors

^b^pTNM: stage classified by the American Joint Committee on Cancer 7th edition TMN system: I, the cancer has grown through the mucosa and has invaded the muscular layer of the colon. No regional lymph node metastasis or distant metastasis exists; II, the cancer has grown through the wall of the colon or through the layers of the muscle to the visceral peritoneum, or has grown into nearby structures. No regional lymph node metastasis or distant metastasis exists; III, metastasis in regional lymph nodes but no distant metastasis; IV, metastasis in regional lymph nodes and distant metastasis

### Evolution in anxiety, depression, general, and specific HRQoL in relation to mortality throughout the 5-year follow-up: survival, non-survival, and normative populations

**Fig. 1:**
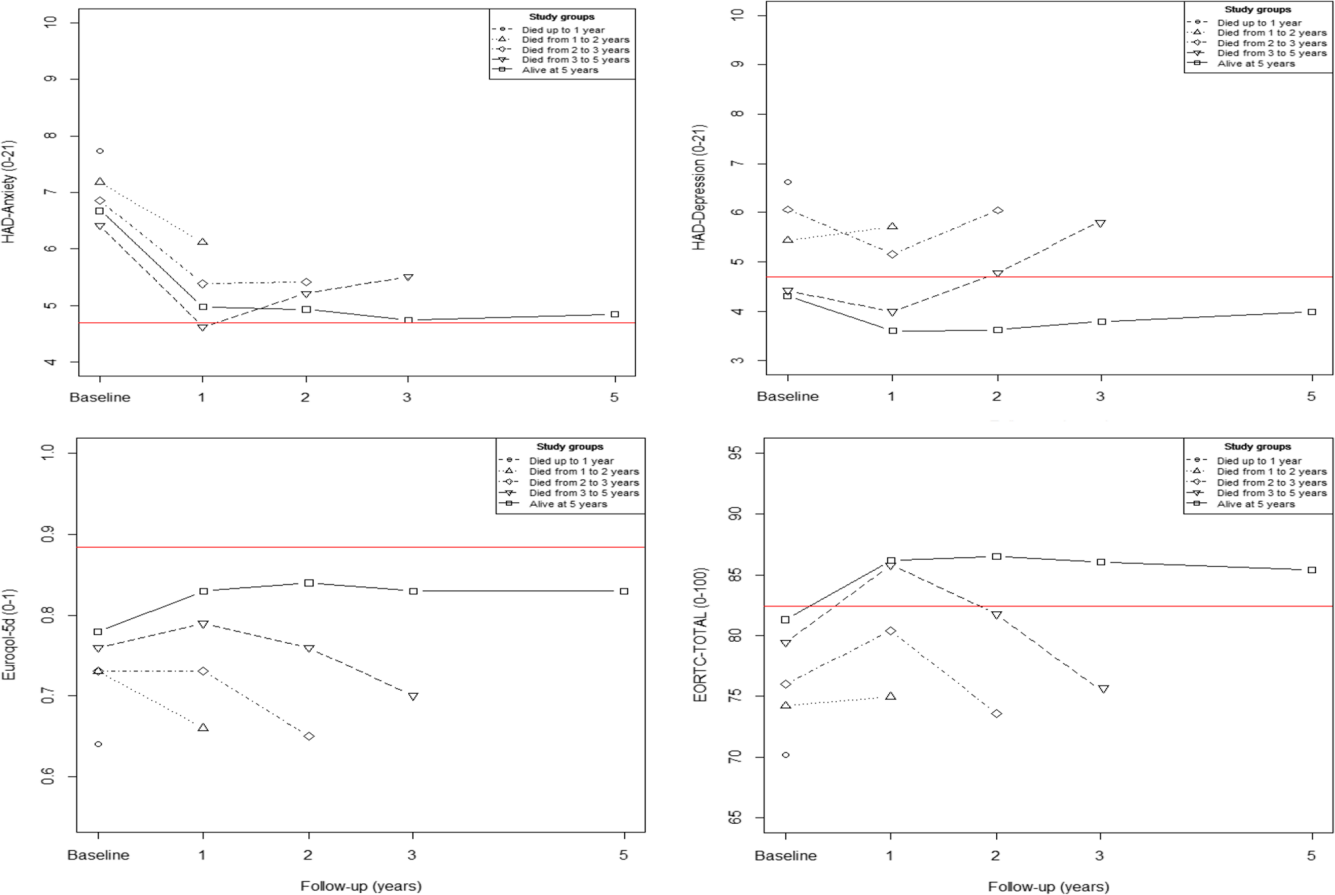
Evolution of HAD anxiety and depression domains, EuroQol-5d-5l, and total EORTC scores in colorectal cancer patients with follow-up until 5 years from index surgical intervention, by vital status time. The red line represents the score for the normative population. Statistical differences between the score in the questionnaires of the normal population and groups of patients according to patients’ vital status throughout evolution. **HAD-Anxiety**: *At baseline:* with all. *At 1-year follow-up:* with the groups of patients who died from 1 to 2 years and those who are alive at 5 years. **HAD-Depression**: *At baseline:* with the group of patients who died up to 1 year; those who died from 2 to 3 years and those who are alive at 5 years. *1 year follow-up*: with those who died from 3 to 5 years and those who are alive at 5 years. *At 2-year follow-up*: with those who are alive at 5 years. *At 3 years follow-up:* with all. *At 5-year follow-up:* with all. **Euroqol-5d**: *At baseline:* with all. *At 1-year follow-up:* with all. *At 2-year follow-up:* with all. *At 3-year follow-up:* with all. *At 5-year follow-up:* with all. **EORTC**: *At baseline:* with all. *At 1-year follow-up:* with all except patients who died from 2 to 3 years. *At 2-years follow-up:* with all, except patients who died from 3 to 5 years. *At 3-year follow-up:* with all. *At 5-year follow-up:* with all

Figure 1 shows the evolution in anxiety, depression, and HRQoL during the 5-year follow-up monitoring of surviving and non-surviving patients with CRC and also in relation to the score of the normative population in these questionnaires. As can be seen, those who died earlier had worse baseline scores and, in both surviving and non-surviving CRC patients, there was a trend over time towards improvement in all variables during the first year after the intervention. This improvement was maintained among survivors in the following years, but worsened among non-survivors as death approached.

Compared to the normative group, the baseline levels of anxiety and HRQoL (both general and specific) were significantly worse (*p*≤ 0.05) among survivors and non-survivors than those in the normative population. However, over time, among survivors, emotional state and specific EORTC-HRQoL returned to the levels of this normative group, with this improvement taking somewhat longer in the case of anxiety, but reached even better levels than the normative population in HAD-depression and specific EORTC-HRQoL. However, they still had a worse general EQ-5D HRQoL. With respect to non-survivors, general EQ-5D HRQoL remained significantly worse over time than those of the normative group, while specific EORTC-HRQoL returned to normative group levels, except in the period before death, when it again became significantly worse. With respect to emotional state, the different survival groups showed no differences over time with the normative population in anxiety, except among the group dying between 1 and 2 years after the intervention, who suffered from higher levels of anxiety in the period immediately prior to death. With regard to depression, we found similar levels to those of the normative group in all groups, except for those dying between 3 and 5 years after the intervention, who had higher levels of depression in the period immediately prior to death.

These results are confirmed in Supplementary Table 1 that shows statistically significant differences in depression and HRQoL throughout the entire follow-up among survivors and non-survivors. Non-survivors had higher depression and worse HRQoL 1 year before death than survivors in the same period of time. In the case of anxiety, these statistically significant differences were found at baseline and 1 year after the intervention.

Table 2 shows the relationship of the scores from the different questionnaires to mortality at each follow-up point in time. Each extra point in HAD-Anxiety and HAD-Depression increased the risk of dying the following year by 7.2% and 12%, respectively. With regard to HRQoL, each 0.10-point decrease in the EQ-5D and 5-point decrease in the EORTC-QLQ-C30 increased the risk of dying the following year by 31% and 22%, respectively.

**Table 2:** Influence among colorectal cancer patients of scores in different health-related quality-of-life scales at each follow-up point on mortality in the following year

	β (s.e.)	OR (95% CI)	*p*-value
HAD-A*	0.07 (0.01)	1.072 (1.046 – 1.099)	<0.0001
HAD-D*	0.11 (0.01)	1.118 (1.092 – 1.145)	<0.0001
EQ-5D-5L Sc1 (0.10 pts)**	0.27 (0.02)	1.312 (1.258 – 1.377)	<0.0001
EORTC-QLQ-C30 total (5 pts)**	0.20 (0.02)	1.221 (1.185 – 1.258)	<0.0001

*β* (s.e.): estimation (standard error); *OR*, odds ratio; *CI*, confidence interval; *%*, percentage; *Sc1*, Scale 1; *Pts*, points; *HAD-A*, Hospital Anxiety and Depression questionnaire’s Anxiety subscale; *HAD-D*, Hospital Anxiety and Depression questionnaire’s Depression subscale; *EQ-5D-5L*, EuroQol-5D-L; *EORTC-QLQ-C30*, The European Organization for Research and Treatment of Cancer Quality of Life Questionnaire Core 30

*Estimations for HAD Anxiety and Depression are calculated for units of increase, which indicates deterioration in anxiety and depression, respectively

**Estimations for EQ-5D-5L and EORTC-QLQ-C30 are calculated for 0.10 and 5 units of decrease, respectively, which indicates deterioration in quality of life

### Significant factors predicting anxiety, depression, general, and specific HRQoL changes at 1 and 5 years after intervention

The univariable analyses are shown in Supplementary Tables 2 and 3 respectively.

Sex and adjuvant chemotherapy were predictors of change from baseline to one year after surgery in anxiety. Females had in mean 0.91 points more of anxiety than males at 1 year and those patients who received adjuvant chemotherapy had 0.27 points more of anxiety than those who did not receive. Change in depression from baseline to 1 year was explained by sex, Charlson score, having complications 1 month after intervention, and having a stoma after main surgery. Females had more depression at 1 year than males and those patients who had complications at 1 month and had a stoma had more depression than those who did not. Sex, Charlson index, and complications during admission and at 1 month after intervention were predictors of HRQoLs from baseline to 1 year in overall (EQ-5D-5L and EORTC-QLQ-C30). Females and those patients who had complications had worse HRQoL at 1 year than males and those patients who did not, respectively. Additionally, for the general HRQoL change from baseline to 1 year (measured by EQ-5D-5L), having a stoma after surgery and needing or receiving social support at baseline resulted in statistically significant predictors of lower HRQoL. In the case of the change of the EORTC-QLQ-C30 from baseline to 1 year, was predicted also by receiving adjuvant chemotherapy and having a family history of CRC. Those patients who received adjuvant chemotherapy and had a family history of CRC had worse HRQoL at 1 year than those who did not (Table 3).

**Table 3:** Associations of demographic, social, and clinical characteristics with changes in HAD-A, HAD-D, EQ-5D-5L, and EORTC-QLQ-C30 1 year after surgery

	HAD-A			HAD-D			EQ-5D-5L			EORTC-QLQ-C30		
	β (s.e.)	CI 95%	*p*-value	β (s.e.)	CI 95%	*p*-value	β (s.e.)	CI 95%	*p*-value	β (s.e.)	CI 95%	*p-*value
Intercept	1.73 (0.15)	1.4–2.01	<0.0001	1.26 (0.12)	1.01–1.50	<0.0001	0.59 (0.02)	0.55–0.62	<0.0001	52.93 (1.41)	50.17–55.70	<0.0001
HAD-A baseline	0.42 (0.02)	0.39–0.45	<0.0001	-	-	-	-	-	-	-	-	-
HAD-D baseline	-	-	-	0.45 (0.02)	0.42–0.48	<0.0001	-	-	-	-	-	-
EQ-5D-5L baseline	-	-	-	-	-	-	0.36 (0.02)	0.33–0.39	<0.0001	-	-	-
EORTC baseline	-	-	-	-	-	-	-	-	-	0.43 (0.02)	0.40–0.46	<0.0001
Sex (female vs male)	0.91 (0.15)	0.62–1.20	<0.0001	0.43 (0.14)	0.15–0.71	0.002	−0.04 (0.01)	−0.05 to −0.02	<0.0001	−2.21 (0.51)	−3.24 to −1.23	<0.0001
Charlson adjusted by age (>5 vs ≤ 5)	-	-	-	0.63 (0.14)	0.35–0.90	<0.0001	−0.03 (0.01)	−0.05 to −0.02	<0.0001	−1.60 (0.52)	−2.63 to −0.57	0.0024
Complications 1 month after intervention	-	-	-	0.54 (0.28)	0.002–1.09	0.05	−0.03 (0.01)	−0.04 to −0.007	0.009	−2.25 (1.05)	−4.30 to −0.19	0.032
Complications during admission (yes vs no)	-	-	-	-	-	-	−0.02 (0.01)	−0.03 to −0.002	0.03	−1.66 (0.74)	−3.10 to −0.22	0.024
Stoma after surgery	-	-	-	0.46 (0.16)	0.14–0.77	0.0046	−0.03 (0.01)	−0.05 to −0.01	0.002			
Adjuvant chemotherapy (yes vs no)	0.27 (0.14)	0.0004–0.55	0.049	-	-	-	-	-	-	–1.27 (0.50)	–2.25 to −0.29	0.0115
Family history of CRC	-	-	-	-	-	-	-	-	-	−1.79 (0.88)	−3.50 to −0.07	0.041
Social support												
Needs and does not receive help (vs does not need)	-	-	-	-	-	-	−0.03 (0.03)	−0.09 to 0.04	0.46	-	-	-
Receives help (vs does not need)	-	-	-	-	-	-	−0.02 (0.01)	−0.03 to −0.001	0.037	-	-	-
*R*^2^		0.31			0.32			0.25		0.33		
*n/N*		1975/2352			1971/2352			1839/2352		1909/2352		

*β (s.e.)*, estimation (standard error); *CI*, confidence interval; *R*^2^, R-squared; *n/N*, sample used for the model/total sample; *HAD-A*, Hospital Anxiety and Depression questionnaire’s Anxiety subscale; *HAD-D*, Hospital Anxiety and Depression questionnaire’s Depression subscale; *EQ-5D-5L*, EuroQol-5D-L; *EORTC-QLQ-C30*, The European Organization for Research and Treatment of Cancer Quality of Life Questionnaire Core 30; *CRC*, colorectal cancer

Change of anxiety from baseline to 5 years was explained by 1-year anxiety, family history of CR neoplasia, complications 1 year after the intervention, sex, age, and pathological TNM (pTNM). Patients with complications 1 year after the intervention had higher levels of anxiety at 5 years than those who did not, as well as females had higher levels of anxiety at 5 years than males. Change of depression from baseline to 5 years was predicted by 1-year depression, Charlson index, family history of neoplasia, complications 1 year after intervention, and needing and receiving social support. Overall, change of HRQoL from baseline to 5 years was predicted by 1-year HRQoL and Charlson index. In CCR-specific HRQoL (measured by EORTC-QLQ-C30), the change from baseline to 5 years was also explained by family CR neoplasia history and complications 1 year after the intervention (Table 4).

**Table 4:** Associations of demographic, social, and clinical characteristics with changes in HAD-A, HAD-D, EQ-5D-5L, and EORTC-QLQ-C30 5 years after surgery

	HAD-A			HAD-D			EQ-5D-5L			EORTC		
	β (s.e.)	CI 95%	*p*-value	β (s.e.)	CI 95%	*p*-value	β (s.e.)	CI 95%	*p*-value	β (s.e.)	CI 95%	*p*-value
Intercept	1.17 (0.17)	0.85–1.50	<0.0001	1.00	0.70–1.30	<0.0001	0.21 (0.02)	0.17–0.25	<0.0001	16.78 (1.87)	13.12–20.44	<0.0001
HAD-A baseline	0.18 (0.02)	0.14–0.22	<0.0001	-	-	-	-	-	-	-	-	-
HAD-D baseline	-	-	-	0.27 (0.02)	0.22–0.31	<0.0001	-	-	-	-	-	-
EQ-5D-5L baseline	-	-	-	-	-	-	0.19 (0.02)	0.15–0.23	<0.0001	-	-	-
EORTC baseline	-	-	-	-	-	-	-	-	-	0.21 (002)	0.17–0.25	<0.0001
HAD-A 1 at T1	0.43 (0.02)	0.38–0.48	<0.0001	-	-	-	-	-	-	-	-	-
HAD-D 1 at T1	-	-	-	0.44 (0.02)	0.39–0.49	<0.0001	-	-	-	-	-	-
EQ-5D-5L at T1	-	-	-	-	-	-	0.57 (0.02)	0.52–0.62	<0.0001	-	-	-
EORTC at T1	-	-	-	-	-	-	-	-	-	0.60 (0.02)	056–0.65	<0.0001
Charlson adjusted by age (>5 vs ≤5)	-	-	-	0.63 (0.18)	0.28–0.98	0.0004	−0.07 (0.01)	−0.08 to −0.05	<0.0001	−3.41 (0.54)	−4.47 to −2.35	<0.0001
Family neoplasia history	−0.40 (0.15)	−0.70 to 0.10	0.0083	−0.45 (0.15)	−0.74 to −0.15	0.004	-	-	-	1.03 (0.51)	0.03–2.04	0.04
Complications at T1 (yes vs no)	0.75 (0.22)	0.33–1.18	0.0005	0.36 (0.17)	0.02–0.70	0.04				−1.63 (0.72)	−3.05 to −0.21	0.025
Sex (female vs male)	0.80 (0.16)	0.50–1.11	<0.0001	-	-	-	-	-	-	-	-	-
Age (>80 vs ≤80)	0.70 (0.26)	0.19–1.22	<0.0073	-	-	-	-	-	-	-	-	-
pTNM												
pTNM III vs pTNM0-I-II	0.39 (0.16)	0.070–0.71	0.017	-	-	-	-	-	-	-	-	-
pTNM IV vs pTNM0-I-II	−0.53 (0.37)	−1.26 to 0.19	0.15	-	-	-	-	-	-	-	-	-
Social support												
Needs and does not receive help (vs does not need)	-	-	-	0.33 (0.67)	−0.99 to 1.65	0.63	-	-	-	-	-	-
Receives help (vs does not need)	-	-	-	0.33 (0.15)	0.02–0.63	0.03	-	-	-	-	-	-
*R*^2^	0.42			0.48			0.46			0.55		
*n/N*	1398/1798			1316/1798			1477/1798			1333/1798		

*β (s.e.)*, estimation (standard error); *CI*, confidence interval; *R*^*2*^, R-squared; *n/N*, sample used for the model/total sample; *HAD-A*, Hospital Anxiety and Depression questionnaire’s Anxiety subscale; *HAD-D*, Hospital Anxiety and Depression questionnaire’s Depression subscale; *EQ-5D-5L*, EuroQol-5D-L; *EORTC-QLQ-C30*, The European Organization for Research and Treatment of Cancer Quality of Life Questionnaire Core 30; *CRC*, colorectal cancer

## Discussion

This is the first prospective cohort study on CRC evaluating the long-term effects of CRC on anxiety, depression, and HRQoL and their relationship to mortality, taking premorbid levels and effects of natural decline into account.

We first found that patient-reported outcomes such as anxiety, depression, and HRQoL, obtained approximately 5 years after the cancer surgery, appear to be similar to the normative group among survivors and worse among non-survivors. Secondly, non-survivors suffered more from symptoms of anxiety and depression and had worse HRQoL over time than survivors and the normative group. Third, higher anxiety, depression, and worse HRQoL were associated with mortality to a statistically significant degree. Fourth, at shorter times from intervention, sex, comorbidities, having complications at 1 month after intervention, having stoma, and receiving adjuvant chemotherapy were statistically significant predictors of changes in both emotional status and HRQoL. At longer times (5 years), comorbidities and having complications 1 month after the intervention were also predictors of emotional status and HRQoL. Baseline anxiety, depression, and HRQoL also predicted short- and long-term anxiety, depression, and HRQoL, respectively.

### Survivors, non-survivors, and normative groups’ anxiety, depression, and HRQoL

A significant proportion of CRC survivors experience clinically significant levels of anxiety and depressive symptoms throughout the trajectory of the illness. [5, 23] However, the trajectories of anxiety and depressive symptoms and health-related quality of life around 2 years after diagnosis have not been well characterized [5] and there are still relatively few studies focusing on long-term CRC survivors. Although this is a fast-emerging area of research, more attention needs to be paid to the psychosocial consequences of CRC. [23] Our study showed significantly worse baseline anxiety and HRQoL levels in CRC survivors and non-surviving patients than among the normative population. With regard to baseline depression, survivors and those who died between 3 and 5 years after diagnosis showed better scores than the normative population. The baseline depression of the remainder of the non-survivors was significantly worse. This is in line with findings by Mols et al. (2013) [24] that showed that cancer (including CRC) patients with depressive symptoms had twice the risk for all-cause mortality, even after adjustments for major clinical predictors.

Over time, in our study, the survivors’ anxiety, depression, and HRQoL levels improved to those of the normative population or even better in depression and specific HRQoL (EORTC). The only index that never attained the level of the normative group was general HRQoL. Non-survivors all scored significantly worse than the normative population. It has been difficult to compare our results with those of the literature, since we were unable to find studies comparing survivors, non-survivors, and respective normative groups.

Varying results are found in the literature regarding anxiety, depression, and HRQoL throughout the trajectory of the illness in long-term survivors. Some concluded that CRC survivors experienced reduced functioning and lower global health/QOL scores, albeit of smaller magnitude, when compared to population controls. [5, 11] However, these results contrast with other studies of CRC survivors apparently showing that they have comparable HRQoL scores and, in some areas, better well-being than their non-cancer controls. [2, 25–27] These also founded a resilient trajectory in the majority of CRC patients using HADS and a clinically significant improvement at 12 months post-surgery in emotional functioning on the EORTC QLQ-C30, after which they remained stable.

### Prospective association of anxiety, depression, and health-related quality of life with mortality

Although the association between depression and mortality has often been investigated in the past, a number of shortcomings remain the studies on depression and mortality. [24] Overall, our results showed that non-survivors had worse anxiety, depression, and HRQoL scores than survivors during the 5-year follow-up, and the baseline scores were worst in those closest to death, worsening as death approached. Our results indicated that higher levels of anxiety and depression, and lower general and specific HRQoL in the previous year were related to subsequent mortality. Our findings are comparable with a meta-analysis from 2009 showing that among cancer patients experiencing depressive symptoms, mortality rates were up to 25% higher (risk ratio (RR) unadjusted=1.25; 95% CI, 1.12–1.40; *p*<0.001). [28]

### Predictors of anxiety, depression, and HRQoL 1 and 5 years after diagnosis

Our data shows that being female, having comorbid conditions, having stoma after surgery, and receiving adjuvant chemotherapy are predictors of worse emotional status and HRQoL 1 year after surgery, whereas requiring social support, having complications during admissions, and having a family history of CRC were predictors only of worse HRQoL. Of these variables, being female, having comorbid conditions, and requiring social support continue to predict poorer mental health 5 years after diagnosis. In addition, new variables emerge as predictors of poorer mental health in the long term, such as, being over 80 years old, having a family history of neoplasia, and having a TNM-III.

As in previous research, [29–31] the most powerful prospective predictor of psychological distress 12 months after diagnosis was previous distress. In our study, baseline and 1-year post-intervention scores on patient-perceived outcome measures predicted later scores. That is, those who, at baseline or 1 year after the intervention, had worse scores for anxiety, depression, and quality of life were more likely to have worse scores later in the third follow-up.

Being female, younger, location of the tumor, advanced disease, [29, 32–35], and higher number of comorbidities [6, 8, 36] have previously been found to be associated with greater anxiety or depression in cancer patients, and we found that female gender and tumor stage were predictors of worse anxiety HAD scores. However, as Gonzalez-Saenz de Tejada et al. (2016) [32] have also shown, our results did not find tumor location as a predictor of changes in anxiety and depression following adjustment for the other variables.

One medical factor expected to result in greater distress is the creation during the main surgical intervention and subsequent presence of a stoma; however, some limited research has examined its relationship to psychological outcomes. [5] One longitudinal study of CRC patients from 3 to 24 months post-surgery found that patients with a stoma had higher levels of depressive symptoms than non-stoma patients. [5, 37] Evidence regarding candidate predictors of low HRQoL was found to be strongest for the presence of a stoma in patients with a high body mass index. [6] HRQoL scores got worse for the survivors between 3 and 5 years. This could be due to increasing age and perhaps the worsening or appearance of new comorbidities.

Our study has some strengths. We were able to monitor a large cohort of CRC patients for as much as 5 years after surgery, collecting information over time with various PROM instruments to develop a full picture of their evolution and recording a large quantity of clinical information from the baseline and follow-up. At the same time, it also has some limitations. As in other cohort studies, one of the main problems was loss of patients and information in the follow-up, which we sought to minimize. In addition, unfortunately, we lack data from a normative population in Spain for some of the PROM tools, though we were able to use data from other European countries.

As conclusions, our study describes the evolution of various PROM tools in CRC patients and identifies some predictors of poor evolution. These findings should enable preventive programs to be established in the follow-up of these patients to reduce their psychological distress and improve their HRQoL as far as possible. This should be performed not only on survivors but also, by different means, among those who have poorer life expectancy.

## Supplementary Information

ESM 1 (DOCX 49 kb)

## Data Availability

Data available upon request.
