# Supplementary material for: Anxiety, depression, health-related quality of life, and mortality among colorectal patients: 5-year follow-up
Source: Support Care Cancer. 2022 Jun 23;30(10):7943–54. doi: 10.1007/s00520-022-07177-1 (PMC9512719; doi:10.1007/s00520-022-07177-1)
Supplement: Supplementary file 1 — (DOCX 49 kb) [file 520_2022_7177_MOESM1_ESM.docx]

**Supplementary** **Table 1.** Descriptive statistics of health-related quality of life (HRQoL) outcomes in follow-up of colorectal cancer

patients to 5 years, by vital status.

|  | T0-T1 | | T1-T2 | | T2-T3 | | T3-T5 | |
| --- | --- | --- | --- | --- | --- | --- | --- | --- |
| Survivors^a^ | **N=2334** | $\bar{\boldsymbol{x}}\boldsymbol{(sd)}$ | **N=2180** | $\bar{\boldsymbol{x}}\boldsymbol{(sd)}$ | **N=2034** | $\bar{\boldsymbol{x}}\boldsymbol{(sd)}$ | **N=1780** | $\bar{\boldsymbol{x}}\boldsymbol{(sd)}$ |
| HAD-A |  | 6.7 (4.6)* |  | 5.0 (4.1)* |  | 5.0 (4.2) | N | 4.7 (4.0) |
| HAD-D |  | 4.5 (4.3)*** |  | 3.7 (4.0)*** |  | 3.8 (4.0)*** |  | 3.8 (4.1)*** |
| EQ-5D-5L |  | 0.77 (0.2)*** |  | 0.82 (0.2)*** |  | 0.83 (0.2)*** |  | 0.83 (0.2)*** |
| EORTC |  | 80.3 (15.9)*** |  | 85.8 (13.6)*** |  | 86.0 (13.7)*** |  | 86.0 (14.2)*** |
| Nonsurvivors | **N=197** | $\bar{\boldsymbol{x}}\boldsymbol{(sd)}$ | **N=154** | $\bar{x} (sd)$ | **N=146** | $\bar{x} (sd)$ | **N=254** | $\bar{x} (sd)$ |
| HAD-A |  | 7.7 (4.8)* |  | 6.6 (5.1)* |  | 5.4 (4.4) |  | 5.5 (4.2) |
| HAD-D |  | 6.6 (5.3)*** |  | 6.2 (5.5)*** |  | 6.1 (5.2)*** |  | 5.8 (4.6)*** |
| EQ-5D-5L |  | 0.64 (0.3)*** |  | 0.64 (0.3)*** |  | 0.65 (0.3)*** |  | 0.70 (0.2)*** |
| EORTC-QLQ-C30 |  | 70.2 (19.7)*** |  | 72.6 (21.0)*** |  | 73.6 (22.2)*** |  | 75.4 (17.8)*** |

T0=Baseline; T1=1 year after admission; T2=2 years after admission; T3= 3 years after admission; T5= 5 years after admission

^a^The answers of the patient-reported outcome measures correspond to the last year of each period, i.e. in period T0-T1, the results of the questionnaires are from T1, in period T1-T2 from T2 and so on

N: Frequency. $\bar{x} \left( sd \right):$ mean (standard deviation). Differences between patients who died and did not die, at the different follow-up points, on the anxiety and depression scales and health-related quality of life scales. *p<0.05, **p<0.01, ***p<0.001. HAD-A: Hospital Anxiety and Depression questionnaire’s Anxiety subscale; HAD-D: Hospital Anxiety and Depression questionnaire’s Depression subscale; EQ-5D-5L:EuroQol-5D-L; EORTC-QLQ-C30: The European Organization for Research and Treatment of Cancer Quality of Life Questionnaire Core 30

**Supplementary Table 2.** Univariable analysis of predictors of changes in patient-reported outcome scales in follow-up of colorectal cancer patients one year from the index surgical intervention. (Adjusted by baseline score).

|  | HAD-Anxiety | | HAD-Depression | | EuroQol-5d | | EORTC-QLQ-C30 Total | |
| --- | --- | --- | --- | --- | --- | --- | --- | --- |
|  | $\beta(s.e.)$ | p-value | $\beta(s.e.)$ | p-value | $\beta(s.e.)$ | p-value | $\beta(s.e.)$ | p-value |
| Total |  |  |  |  |  |  |  |  |
| Gender (Female *vs.* Male) | 0.91 (0.15) | <0.0001 | 0.36 (0.14) | 0.0112 | -0.04 (0.01) | <0.0001 | -2.04 (0.51) | <0.0001 |
| Age (>80 *vs.* ≤80) | -0.08 (0.22) | 0.7105 | 0.43 (0.21) | 0.0446 | -0.04 (0.01) | 0.0007 | -0.91 (0.77) | 0.2328 |
| Hemoglobin (≥10 *vs.* <10) | -0.23 (0.22) | 0.2908 | -0.11 (0.21) | 0.6145 | -0.002 (0.01) | 0.8492 | 1.30 (0.77) | 0.0894 |
| Smoking habit |  |  |  |  |  |  |  |  |
| Smoker *vs.* Never smoked | -0.15 (0.22) | 0.4987 | -0.27 (0.22) | 0.2034 | 0.02 (0.01) | 0.1462 | -0.80 (0.76) | 0.2945 |
| Ex-smoker *vs.* Never smoked | -0.49 (0.15) | 0.0013 | -0.33 (0.15) | 0.0221 | 0.02 (0.01) | 0.0059 | 0.26 (0.52) | 0.6152 |
| Charlson index adjusted by age (>5 *vs.* ≤5) | -0.12 (0.15) | 0.4068 | 0.57 (0.14) | <0.0001 | -0.03 (0.01) | <0.0001 | -0.84 (0.50) | 0.0926 |
| Charlson index (≥4 *vs.* <4) | -0.04 (0.17) | 0.8170 | 0.45 (0.17) | 0.0071 | -0.03 (0.01) | 0.0002 | -0.66 (0.59) | 0.2634 |
| Family history neoplasm (Yes *vs.* no) | 0.15 (0.15) | 0.3389 | -0.10 (0.15) | 0.4967 | 0.01 (0.01) | 0.2699 | -0.07 (0.52) | 0.8895 |
| Family history of CRC (Yes *vs.* no) | 0.23 (0.26) | 0.3796 | 0.32 (0.25) | 0.1972 | -0.005 (0.01) | 0.7263 | -1.61 (0.88) | 0.0674 |
| ASA (IV *vs.* I,II,III) | -0.52 (0.39) | 0.1848 | 0.20 (0.38) | 0.5952 | -0.04 (0.02) | 0.0610 | -0.13 (1.36) | 0.9263 |
| Type of intervention (Urgent *vs.* programmed) | -0.87 (0.43) | 0.0454 | -0.53 (0.42) | 0.2008 | 0.05 (0.02) | 0.0267 | 4.47 (1.49) | 0.0027 |
| Type of surgery |  |  |  |  |  |  |  |  |
| Laparoscopy *vs.* Open surgery | -0.08 (0.15) | 0.5931 | -0.12 (0.14) | 0.3762 | 0.01 (0.01) | 0.0567 | 0.46 (0.50) | 0.3599 |
| TEM *vs.* Open surgery | 0.54 (0.69) | 0.4376 | 0.08 (0.66) | 0.9033 | 0.03 (0.04) | 0.4148 | 1.16 (2.33) | 0.6191 |
| Invasion to other organs |  |  |  |  |  |  |  |  |
| 1 *vs.* 0 | 0.13 (0.40) | 0.7497 | -0.01 (0.38) | 0.9783 | 0.01 (0.02) | 0.6566 | -1.03 (1.36) | 0.4516 |
| >1 *vs.* 0 | -0.44 (1.05) | 0.6717 | 0.02 (1.01) | 0.9833 | 0.08 (0.05) | 0.1592 | 2.84 (3.74) | 0.4467 |
| Intra-surgery complications (Yes *vs.* no) | -0.01 (0.24) | 0.9732 | 0.27 (0.23) | 0.2380 | -0.02 (0.01) | 0.1960 | -1.18 (0.82) | 0.1515 |
| pTNM^a^ |  |  |  |  |  |  |  |  |
| III *vs.* 0,I,II | 0.02 (0.15) | 0.9135 | 0.02 (0.15) | 0.8714 | -0.01 (0.01) | 0.2676 | -0.13 (0.53) | 0.8033 |
| IV *vs.* 0,I,II | -0.08 (0.27) | 0.7620 | 0.05 (0.26) | 0.8432 | -0.005 (0.01) | 0.7205 | -1.42 (0.93) | 0.1257 |
| Vascular, perineural or lymphatic invasion (2,3 *vs.* none,1) | 0.41 (0.24) | 0.0876 | 0.28 (0.23) | 0.2272 | -0.01 (0.01) | 0.3063 | -0.65 (0.82) | 0.4228 |
| Result of the surgery (R2 *vs.* R0,R1) | -0.19 (0.52) | 0.7094 | 0.35 (0.50) | 0.4864 | -0.02 (0.03) | 0.4133 | -1.16 (1.78) | 0.5158 |
| Log lymph nodes ratio |  |  |  |  |  |  |  |  |
| -1.36< ≤-0.53 *vs.* ≤-1.36 | -0.05 (0.25) | 0.8275 | -0.02 (0.24) | 0.9241 | -0.02 (0.01) | 0.1863 | -0.16 (0.85) | 0.8473 |
| -0.53< *vs.* ≤-1.36 | 0.09 (0.29) | 0.7420 | 0.22 (0.27) | 0.4159 | -0.02 (0.01) | 0.1184 | -0.79 (0.97) | 0.4141 |
| Tumor differentiation (High *vs.* Low) | 0.30 (0.23) | 0.1888 | 0.01 (0.22) | 0.9785 | -0.003 (0.01) | 0.7793 | -1.06 (0.76) | 0.1646 |
| Neoadjuvant chemotherapy (Yes *vs.* no) | 0.20 (0.19) | 0.3098 | 0.29 (0.18) | 0.1197 | -0.01 (0.01) | 0.3919 | -0.55 (0.66) | 0.3991 |
| Adjuvant chemotherapy (Yes *vs.* no) | 0.29 (0.14) | 0.0410 | -0.10 (0.14) | 0.4684 | 0.002 (0.01) | 0.7744 | -0.91 (0.48) | 0.0576 |
| Complications during admission (Yes *vs.* no) | -0.004 (0.14) | 0.9744 | 0.24 (0.14) | 0.0873 | -0.02 (0.01) | 0.0053 | -0.92 (0.49) | 0.0608 |
| Infectious (Yes *vs.* no) | 0.09 (0.18) | 0.6182 | 0.22 (0.17) | 0.2115 | -0.01 (0.01) | 0.2119 | -0.56 (0.62) | 0.3596 |
| Surgical (Yes *vs.* no) | -0.007 (0.21) | 0.9729 | 0.42 (0.20) | 0.0408 | -0.01 (0.01) | 0.2197 | -1.53 (0.73) | 0.0374 |
| Medical (Yes *vs.* no) | -0.003 (0.18) | 0.9886 | 0.05 (0.17) | 0.7602 | -0.01 (0.01) | 0.2135 | -0.21 (0.61) | 0.7312 |
| Complications up to 1 month (Yes *vs.* no) | 0.53 (0.19) | 0.0043 | 0.44 (0.18) | 0.0139 | -0.03 (0.01) | 0.0049 | -2.39 (0.63) | 0.0002 |
| Infectious (Yes *vs.* no) | 0.57 (0.29) | 0.0494 | 0.58 (0.28) | 0.0389 | -0.02 (0.02) | 0.1371 | -1.97 (0.99) | 0.0474 |
| Surgical (Yes *vs.* no) | 0.66 (0.31) | 0.0348 | 0.58 (0.30) | 0.0526 | -0.04 (0.02) | 0.0209 | -2.36 (1.05) | 0.0249 |
| Medical (Yes *vs.* no) | 0.20 (0.43) | 0.6476 | 0.17 (0.42) | 0.6745 | 0.01 (0.02) | 0.6345 | -1.20 (1.48) | 0.4170 |
| Barthel index at baseline* | -0.002 (0.01) | 0.7514 | -0.01 (0.01) | 0.1157 | 0.002 (0) | <0.0001 | 0.03 (0.02) | 0.1705 |
| Situation at home |  |  |  |  |  |  |  |  |
| Lives alone *vs.* Lives accompanied | 0.50 (0.21) | 0.0169 | 0.39 (0.20) | 0.0510 | -0.04 (0.01) | 0.0004 | -1.61 (0.72) | 0.0248 |
| Lives in a nursing home *vs.* Lives accompanied | 1.11 (1.28) | 0.3872 | 2.47 (1.24) | 0.0464 | -0.10 (0.07) | 0.1330 | -4.22 (4.31) | 0.3275 |
| Social support |  |  |  |  |  |  |  |  |
| Needs and does not receive help *vs.* Does not need help | 0.59 (0.68) | 0.3857 | 0.01 (0.65) | 0.9858 | -0.03 (0.03) | 0.4076 | -1.62 (2.35) | 0.4906 |
| Receives help *vs.* Does not need help | 0.03 (0.15) | 0.8464 | 0.12 (0.14) | 0.4128 | -0.02 (0.01) | 0.0015 | 0.05 (0.52) | 0.9262 |
| Stoma (Yes *vs.* no) | 0.19 (0.17) | 0.2692 | 0.40 (0.16) | 0.0128 | -0.03 (0.01) | 0.0028 | -0.57 (0.58) | 0.3263 |

β (s.e.): estimation (standard error); HAD-A: Hospital Anxiety and Depression questionnaire’s Anxiety subscale; HAD-D: Hospital Anxiety and Depression questionnaire’s Depression subscale; EQ-5D-5L:EuroQol-5D-L; EORTC-QLQ-C30: The European Organisation for Research and Treatment of Cancer Quality of Life Questionnaire Core 30, total score; CRC: colo-rectal cancer

**^a^** pTNM: stage classified by the American Joint Committee on Cancer 7th edition TMN system: I, the cancer has grown *through the mucosa and has invaded the muscular layer of the colon. No regional lymph node metastasis or distant metastasis exists; II the cancer has grown through the wall of the colon, or through the layers of the muscle to the visceral peritoneum, or has grown into nearby structures. No regional lymph node metastasis or distant metastasis exists; III, metastasis in regional lymph nodes but no distant metastasis; IV metastasis in regional lymph nodes and distant metastasis*.

**Supplementary Table 3.** Univariable analysis of predictors of changes in HRQoL scales in the follow up of colorectal cancer patients at five years from the index surgical intervention. (Adjusted by baseline and one year follow-up scores)

|  | HAD-Anxiety | | HAD-Depression | | EuroQol-5D-5L | | EORTC-Total | |
| --- | --- | --- | --- | --- | --- | --- | --- | --- |
|  | $\beta(s.e.)$ | p-value | $\beta(s.e.)$ | p-value | $\beta(s.e.)$ | p-value | $\beta(s.e.)$ | p-value |
| Total |  |  |  |  |  |  |  |  |
| Gender (Female *vs.* Male) | 0.79 (0.15) | <0.0001 | -0.04 (0.15) | 0.7785 | -0.01 (0.01) | 0.1322 | 0.40 (0.51) | 0.4276 |
| Age (>80 *vs.* ≤80) | 0.76 (0.26) | 0.0028 | 1.70 (0.25) | <0.0001 | -0.07 (0.01) | <0.0001 | -3.75 (0.86) | <0.0001 |
| Hemoglobin (≥10 *vs.* <10) | 0.21 (0.24) | 0.3931 | 0.26 (0.24) | 0.2748 | 0.01 (0.01) | 0.6977 | 0.15 (0.81) | 0.8521 |
| Smoking habit |  |  |  |  |  |  |  |  |
| Smoker *vs.* Never smoked | -0.36 (0.22) | 0.1077 | -0.29 (0.22) | 0.1802 | 0.02 (0.01) | 0.0392 | 0.76 (0.74) | 0.3055 |
| Ex-smoker *vs.* Never smoked | -0.48 (0.16) | 0.0027 | -0.07 (0.15) | 0.6601 | 0.01 (0.01) | 0.1823 | 0.21 (0.52) | 0.6930 |
| Charlson index adjusted by age (>5 *vs.* ≤5) | 0.34 (0.15) | 0.0282 | 0.97 (0.15) | <0.0001 | -0.07 (0.01) | <0.0001 | -3.37 (0.50) | <0.0001 |
| Charlson index (≥4 *vs.* <4) | 0.21 (0.19) | 0.2743 | 0.42 (0.19) | 0.0235 | -0.05 (0.01) | <0.0001 | -2.56 (0.63) | <0.0001 |
| Family history neoplasm (Yes *vs.* no) | -0.37 (0.16) | 0.0166 | -0.54 (0.15) | 0.0003 | 0.02 (0.01) | 0.0077 | 1.44 (0.52) | 0.0055 |
| Family history of CRC (Yes *vs.* no) | -0.16 (0.26) | 0.5349 | -0.40 (0.25) | 0.1198 | 0.01 (0.01) | 0.4960 | 0.91 (0.86) | 0.2920 |
| ASA (IV *vs.* I,II,III) | 0.20 (0.46) | 0.6651 | 0.93 (0.45) | 0.0380 | -0.09 (0.02) | 0.0003 | -1.56 (1.55) | 0.3143 |
| Type of intervention (Urgent *vs.* Programmed) | -0.01 (0.52) | 0.9863 | -0.59 (0.50) | 0.2416 | 0.07 (0.03) | 0.0081 | 4.65 (1.71) | 0.0065 |
| Type of surgery |  |  |  |  |  |  |  |  |
| Laparoscopy *vs.* Open surgery | -0.31 (0.15) | 0.0450 | -0.27 (0.15) | 0.0683 | 0.002 (0.01) | 0.7646 | 0.97 (0.51) | 0.0570 |
| TEM *vs.* Open surgery | 0.39 (0.68) | 0.5636 | -0.40 (0.66) | 0.5443 | -0.01 (0.04) | 0.8417 | 3.86 (2.21) | 0.0810 |
| Invasion to other organs |  |  |  |  |  |  |  |  |
| 1 *vs.* 0 | -0.47 (0.45) | 0.3001 | -0.17 (0.44) | 0.7038 | 0.03 (0.02) | 0.2566 | 1.98 (1.50) | 0.1862 |
| >1 *vs.* 0 | -1.17 (1.16) | 0.3141 | -1.75 (1.13) | 0.1227 | -0.06 (0.06) | 0.3385 | -0.69 (4.13) | 0.8669 |
| Intrasurgical complications (Yes *vs.* no) | 0.14 (0.26) | 0.5888 | 0.09 (0.25) | 0.7232 | -0.02 (0.01) | 0.1421 | -1.06 (0.84) | 0.2067 |
| pTNM |  |  |  |  |  |  |  |  |
| III *vs.* 0,I,II | 0.37 (0.16) | 0.0226 | 0.24 (0.16) | 0.1202 | 0.02 (0.01) | 0.0376 | 1.20 (0.53) | 0.0241 |
| IV *vs.* 0,I,II | -0.41 (0.35) | 0.2470 | -0.55 (0.35) | 0.1140 | 0.02 (0.02) | 0.2209 | -0.53 (1.17) | 0.6505 |
| Vascular, perineural or lymphatic invasion (2,3 *vs.* none,1) | 0.37 (0.28) | 0.1902 | 0.08 (0.27) | 0.7575 | 0.01 (0.01) | 0.6130 | 0.01 (0.92) | 0.9888 |
| Result of the surgery (R2 *vs.* R0,R1) | -0.07 (0.95) | 0.9447 | -0.61 (0.91) | 0.5085 | 0.05 (0.05) | 0.3081 | 1.74 (3.08) | 0.5725 |
| Log lymph nodes ratio |  |  |  |  |  |  |  |  |
| -1.36< ≤-0.53 *vs.* ≤-1.36 | 0.28 (0.28) | 0.3186 | -0.07 (0.28) | 0.8080 | 0.01 (0.02) | 0.4087 | -0.44 (0.95) | 0.6415 |
| -0.53< *vs.* ≤-1.36 | -0.12 (0.34) | 0.7305 | -0.59 (0.34) | 0.0801 | 0.03 (0.02) | 0.0586 | 2.56 (1.11) | 0.0213 |
| Tumor differentiation (High *vs.* Low) | 0.21 (0.25) | 0.3913 | 0.15 (0.24) | 0.5359 | -0.01 (0.01) | 0.6628 | -0.21 (0.82) | 0.7947 |
| Neoadjuvant chemotherapy (Yes *vs.* no) | -0.01 (0.20) | 0.9663 | -0.26 (0.19) | 0.1859 | 0.004 (0.01) | 0.6857 | -1.04 (0.65) | 0.1117 |
| Adjuvant chemotherapy (Yes *vs.* no) | 0.22 (0.15) | 0.1298 | -0.13 (0.14) | 0.3747 | 0.03 (0.01) | <0.0001 | 0.96 (0.48) | 0.0454 |
| Complications during admission (Yes *vs.* no) | 0.06 (0.15) | 0.6989 | 0.11 (0.15) | 0.4561 | -0.02 (0.01) | 0.0352 | -0.28 (0.50) | 0.5659 |
| Infectious (Yes *vs.* no) | -0.10 (0.19) | 0.5927 | -0.16 (0.19) | 0.4008 | -0.01 (0.01) | 0.4146 | -0.53 (0.64) | 0.4132 |
| Surgical (Yes *vs.* no) | -0.13 (0.22) | 0.5726 | -0.04 (0.22) | 0.8532 | -0.01 (0.01) | 0.6029 | 0.85 (0.75) | 0.2613 |
| Medical (Yes *vs.* no) | 0.11 (0.19) | 0.5694 | 0.23 (0.19) | 0.2250 | -0.02 (0.01) | 0.0673 | -1.08 (0.63) | 0.0879 |
| Complications up to 1 month (Yes *vs.* no) | 0.002 (0.20) | 0.9918 | -0.15 (0.19) | 0.4396 | 0.002 (0.01) | 0.8141 | 0.64 (0.65) | 0.3252 |
| Infectious (Yes *vs.* no) | -0.04 (0.31) | 0.8985 | -0.02 (0.30) | 0.9445 | 0.01 (0.02) | 0.6066 | 0.16 (1.02) | 0.8769 |
| Surgical (Yes *vs.* no) | -0.09 (0.32) | 0.7681 | -0.35 (0.31) | 0.2668 | -0.01 (0.02) | 0.7127 | 0.05 (1.04) | 0.9629 |
| Medical (Yes *vs.* no) | -0.44 (0.46) | 0.3402 | -0.09 (0.45) | 0.8367 | -0.05 (0.02) | 0.0497 | 0.23 (1.56) | 0.8809 |
| Complications up to 1 year (Yes *vs.* no) | 0.62 (0.17) | 0.0003 | 0.40 (0.17) | 0.0170 | -0.01 (0.01) | 0.1698 | -1.27 (0.57) | 0.0266 |
| Infectious (Yes *vs.* no) | 0.17 (0.40) | 0.6722 | -0.17 (0.39) | 0.6612 | 0 (0.02) | 0.9667 | -0.22 (1.37) | 0.8700 |
| Surgical (Yes *vs.* no) | 0.57 (0.21) | 0.0078 | 0.17 (0.21) | 0.4258 | -0.01 (0.01) | 0.2159 | -1.63 (0.70) | 0.0203 |
| Medical (Yes *vs.* no) | 0.64 (0.34) | 0.0626 | 0.25 (0.33) | 0.4623 | -0.04 (0.02) | 0.0540 | -0.79 (1.13) | 0.4846 |
| Barthel index at baseline* | -0.01 (0.01) | 0.2387 | -0.01 (0.01) | 0.1550 | 0.001 (0) | 0.0018 | 0.03 (0.03) | 0.2685 |
| Situation at home |  |  |  |  |  |  |  |  |
| Lives alone *vs.* Lives accompanied | 0.11 (0.22) | 0.6111 | 0.46 (0.22) | 0.0326 | -0.01 (0.01) | 0.5279 | -0.78 (0.74) | 0.2899 |
| Lives in a nursing home *vs.* Lives accompanied | 0.70 (2.84) | 0.8047 | 0.28 (2.77) | 0.9208 | 0.002 (0.15) | 0.9880 | 0.60 (9.28) | 0.9482 |
| Social support |  |  |  |  |  |  |  |  |
| Needs and does not receive help *vs.* Does not need help | -0.51 (0.68) | 0.4537 | 0.12 (0.66) | 0.8595 | 0.01 (0.04) | 0.8580 | 0.12 (2.28) | 0.9569 |
| Receives help *vs.* Does not need help | 0.40 (0.15) | 0.0078 | 0.46 (0.15) | 0.0020 | -0.02 (0.01) | 0.0134 | -0.89 (0.52) | 0.0870 |
| Stoma (Yes *vs.* no) | 0.07 (0.14) | 0.5920 | 0.03 (0.13) | 0.8371 | -0.002 (0.01) | 0.7686 | -0.51 (0.45) | 0.2547 |

β (s.e.): estimation (standard error); HAD-A: Hospital Anxiety and Depression questionnaire’s Anxiety subscale; HAD-D: Hospital Anxiety and Depression questionnaire’s Depression subscale; EQ-5D-5L:EuroQol-5D-L; EORTC-QLQ-C30 Total: The European Organisation for Research and Treatment of Cancer Quality of Life Questionnaire Core 30, total score; CRC: colo-rectal cancer

**^a^** pTNM: stage classified by the American Joint Committee on Cancer 7th edition TMN system: I, the cancer has grown *through the mucosa and has invaded the muscular layer of the colon. No regional lymph node metastasis or distant metastasis exists; II the cancer has grown through the wall of the colon, or through the layers of the muscle to the visceral peritoneum, or has grown into nearby structures. No regional lymph node metastasis or distant metastasis exists; III, metastasis in regional lymph nodes but no distant metastasis; IV metastasis in regional lymph nodes and distant metastasis*.

**Supplementary Figure 1**. Flowchart describing the cohort evolution during the five years of follow-up

**Recruited patients with colon or rectum cancer**

**N = 3602**

**Excluded patients: 853 (23.68%)**

**Exclusion cause:**

Poor condition/terminal 33 (3.87%)

Refusal to participate 512 (60.02%)

Language problems 4 (0.47%)

Sensory problems/Institutionalized 13 (1.52%)

Cancer in situ 113 (13.25%)

Inoperable 46 (5.39%)

Recurrence of the tumor 60 (7.03%)

Pre-intervention death 35 (4.10%)

No intervention 12 (1.41%)

Referred to another hospital 16 (1.88%)

Not specified 9 (1.06%)

**Fullfilled inclusión criteria**

**N = 2749**

Patients’ questionnaires at baseline

**N = 2228**

Patients’ questionnaires at 1 year of follow-up

**N = 1691**

Patients’ questionnaires at 2 years of follow-up

**N = 1488**

Patients’ questionnaires at 3 years of follow-up

**N = 1243**

Patients’ questionnaires at 5 years of follow-up

**N = 1116**

**Losses up to 1 year: 537 (24.10%)**

Died during the admission 21 (3.91%)

Died up to 1 month 7 (1.30%)

Died up to 1 year 128 (23.84%)

Lost to follow-up 381 (70.95%)

**Losses from 1 to 2 years: 203 (12.00%)**

Died up to 2 years 124 (61.08%)

Lost to follow-up 79 (38.92%)

**Losses from 2 to 3 years: 245 (16.47%)**

Patients from hospitals with no follow-up at 3 and 5 years 117 (47.76%)

Died up to 3 years 52 (21.22%)

Lost to follow-up 76 (31.02%)

**Losses from 3 to 5 years: 127 (10.22%)**

Died up to 5 years 92 (72.44%)

Lost to follow-up 35 (27.56%)
